# Effects of *passiflora incarnata* and midazolam for control of anxiety in patients undergoing dental extraction

**DOI:** 10.4317/medoral.21140

**Published:** 2016-12-06

**Authors:** Liliane-Poconé Dantas, Artur de Oliveira-Ribeiro, Liane-Maciel de Almeida-Souza, Francisco-Carlos Groppo

**Affiliations:** 1 Msc, Federal University of Sergipe, Aracaju, Sergipe, Brasil; 2 PhD, Federal University of Sergipe, Aracaju, Sergipe, Brasil; 3 PhD, Dentistry College of Piracicaba , FOP, UNICAMP, Piracicaba, São Paulo, Brasil

## Abstract

**Background:**

Anxiety symptoms are frequently observed in dental patients, whether they are undergoing simple or more invasive procedures such as surgery. This research aimed to compare the effects of *Passiflora incarnata* and midazolam for the control of anxiety in patients undergoing mandibular third molar extraction.

**Material and Methods:**

Forty volunteers underwent bilateral extraction of their mandibular third molars in a randomized, controlled, double-blind, crossover clinical trial. *Passiflora incarnata* (260 mg) or midazolam (15 mg) were orally administered 30 minutes before surgery. The anxiety level of participants was evaluated by questionnaires and measurement of physical parameters, including heart rate (HR), blood pressure (BP), and oxygen saturation (SpO2).

**Results:**

Considering each procedure independently, there were no significant differences between the protocols in BP, HR, and SpO2. Over 70% of the volunteers responded that they felt quiet or a little anxious under both protocols. With midazolam, 20% of the participants reported amnesia (not remembering anything at all), while *Passiflora* showed little or no ability to interfere with memory formation.

**Conclusions:**

*Passiflora incarnata* showed an anxiolytic effect similar to midazolam, and was safe and effective for conscious sedation in adult patients who underwent extraction of their mandibular third molars.

**Key words:**Passiflora incarnata, midazolam, anxiety, oral surgery.

## Introduction

Anxiety can be identified in most patients seeking dental care by observing behavior and recognizing physiological signs of anxiety, such as pupil dilation, pallor, excessive sweating, increased blood pressure and heart rate, tremors, dizziness, dry mouth, weakness, and difficulty breathing. Dental anxiety, a feeling of anxiety or fear at the prospect of dental treatment, is recognized as one of the greatest challenges to professional care (1). A Brazilian study (2) that assessed the prevalence of anxiety during dental treatment using the Dental Anxiety Scale (3) found that 92.4% of subjects had some degree of anxiety. Sedation reduces anxiety, irritability, or agitation through the administration of sedatives to facilitate planned dental procedures. The goals of sedation are to allow dentists to work effectively and to help patients remain as relaxed and comfortable as possible (4).

Oral benzodiazepines have a large margin of clinical safety and are easy to administer. Despite their low toxicity, patients may experience adverse reactions such as a rash, nausea, or headache. Benzodiazepines potentiate the effect of ethanol and may promote a paradoxical reaction. In addition, sedation with benzodiazepines requires that patients be accompanied to appointments with the recommendation not to operate motor vehicles or hazardous machinery for the duration of the drug’s pharmacological effects. Among the benzodiazepines, midazolam is most commonly used for sedation during dental treatments (5-7).

Phytotherapy is among the most popular complementary therapies, and depression and anxiety are important indications for its use. Besides having a lower cost, phytotherapy carries a lower risk of collateral effects and addiction (8,9). The *Passiflora incarnata* plant, belonging to the *Passifloraceae* family and commonly known as passion fruit, is widespread in tropical areas around the world and used in traditional medicine for the treatment of anxiety, nervousness, and neuralgia (10).

Up to now, only 2 published randomized clinical trials (11,12) have reported the anxiolytic effects of preoperative *Passiflora incarnata*. However, neither study involved extraction of the third molars. Although *Passiflora incarnata* is traditionally used as a mild sedative and anxiolytic worldwide, there is a paucity of evidence from randomized clinical trials to demonstrate this activity. Thus, this research aimed to compare the effect of *Passiflora incarnata* with midazolam in the control of anxiety in patients undergoing extraction of mandibular third molars.

## Material and Methods

The Research Ethics Committee of the Federal University of Sergipe approved this project (protocol number 06509812.6.0000.0058). The study was designed as a controlled, randomized, double-blind, crossover clinical trial. Participants signed an agreement of free and informed consent after receiving a detailed explanation of the general objectives of the study and their respective procedures. One researcher collected data between November 2012 and September 2013.

Forty volunteer patients from the Department of Dentistry, Federal University of Sergipe, were selected after diagnosis for bilateral extraction of impacted mandibular third molars. Volunteers were evaluated for asymptomatic third molars, surgical positions, and similar difficulties, according to Pell and Gregory’s classification system. Exclusion criteria included: age younger than 18 years; American Society of Anesthesiology (ASA) classification III or IV; history of using medicines for pain or anxiety in the 15 days before the study; history of hypersensitivity to drugs, substances, or materials used in this experiment; pregnancy or lactation; and history of pericoronitis.

The participants received 15 mg of midazolam (1 pill) or 260 mg of *Passiflora incarnata* (1 pill) administered orally 30 minutes before the start of the surgical procedure. In a crossover design, participants were randomly assigned an extraction side (right or left) and a protocol (midazolam or *Passiflora*) at the first procedure. The researcher delivered the drugs to the participants in encoded form as “Protocol 1” (midazolam) or “Protocol 2” (*Passiflora*). The protocols were not identified until the end of the experiment; therefore, the participants, the surgeon, and the statistician were blinded to the pharmacological treatments being employed. In order to prevent pain and postsurgical edema, a single dose of intramuscular dexamethasone (8 mg) was administered 30 minutes before surgery.

Surgical procedures were performed in 2 sessions, one for each side of the mandible. The side to be operated on in the first session was randomly selected. A single maxillofacial surgeon at the Department of Dentistry, Federal University of Sergipe, performed the extractions. The minimum interval between the first and second surgery was 15 days, and the maximum interval was 30 days.

The anxiety level of the subjects was assessed in 3 distinct phases using questionnaires and measurement of physical parameters. Phase I (baseline) occurred at the initial consultation, one week before the scheduled day of the first intervention. The Corah Dental Anxiety Scale (3) was used to classify participants according to their degree of anxiety. With the participants resting for 5 minutes with their arms positioned at heart level, arterial blood pressure (BP) was assessed using a wrist monitor (Techline, São Paulo, SP, Brazil). Heart rate (HR) and blood oxygen saturation (SpO2) were measured using a digital pulse oximeter (More Fitness, São Paulo, SP, Brazil). All measurements were made by the same researcher and the apparatus was properly calibrated. These measurements served as baseline data for the physical parameters used to evaluate anxiety.

In Phase II (day of surgery), the investigator and the operator responsible for the surgery assessed the degree of anxiety by independently administering the same questionnaire to the participant at the end of the surgical procedure. In addition, the researcher measured BP, HR, and SpO2 at the following time points during the procedure: 30 minutes after drug administration, administration of local anesthesia, incision, extraction of the tooth, and suturing.

In Phase III (return visits), sutures were removed and volunteers returned on the day after surgery to record a self-assessment that asked about the treatment experience, the occurrence of anterograde amnesia, collateral effects, and surgical preference (first or second surgery).

The Mann-Whitney test, Kruskal-Wallis test, multivariate analysis of variance (ANOVA), 2-way ANOVA, chi-square test, and Fisher exact test were used to perform the statistical analysis. A significance level of 5% was adopted for all tests. All data were analyzed with OriginPro 8.0 SRO software (OriginLab Corporation, Northampton, MA, USA).

## Results

Twenty-seven out of 40 volunteers (67.5%) were female, and there were significantly (Fisher exact test, *p* = 0.0398) more women in the sample. However, mean age did not differ significantly (Mann-Whitney test, *p* = 0.6302) between men (23.4 ± 5.2 years) and women (24.2 ± 4.5 years).

 Table 1 shows the influence of gender on the anxiety level of the volunteers. A statistically significant difference (Mann-Whitney test, *p* = 0.0090) was observed between genders, with women having higher levels of anxiety than men.

**Table 1:**
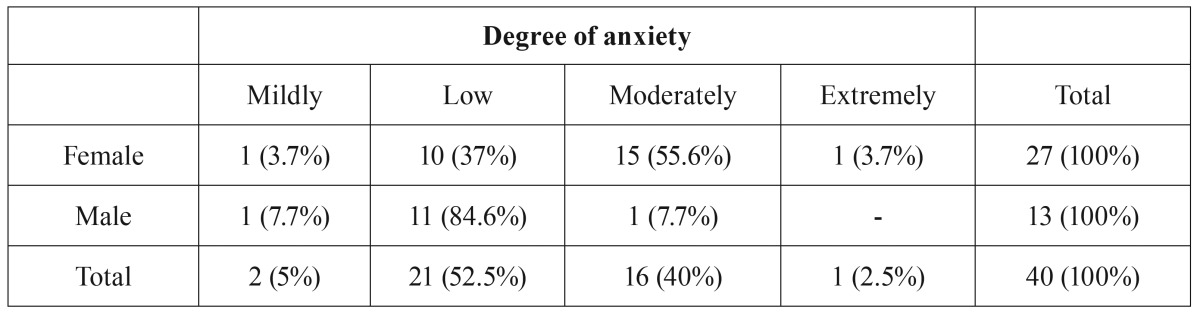
Influence of gender on participants’anxiety levels.

To understand the effect of age on the degree of anxiety, data were divided into 3 groups. The previously described scores were used for comparison of anxiety and age. No statistically significant differences (Kruskal-Wallis, *p* = 0.0891) were found between ages, indicating that age did not influence anxiety levels.

Multivariate ANOVA did not reveal a statistically significant difference in systolic BP based on the side of the extraction (*p* = 0.77250) or the order of surgery (0.50259), independent of the observation period. Similarly, the operated side and the order of surgery did not influence the minimum pressure (side: *p* = 0.97070; order of surgery: *p* = 0.27183), HR (side: *p* = 0.33587; order of surgery: *p* = 0.82525), and SpO2 (side: *p* = 0.49356; order of surgery: *p* = 0.85607). Thus, in analyzing the effect of Protocols 1 and 2, the side of the extraction and the order of surgery were not considered.

**Figure 1:**
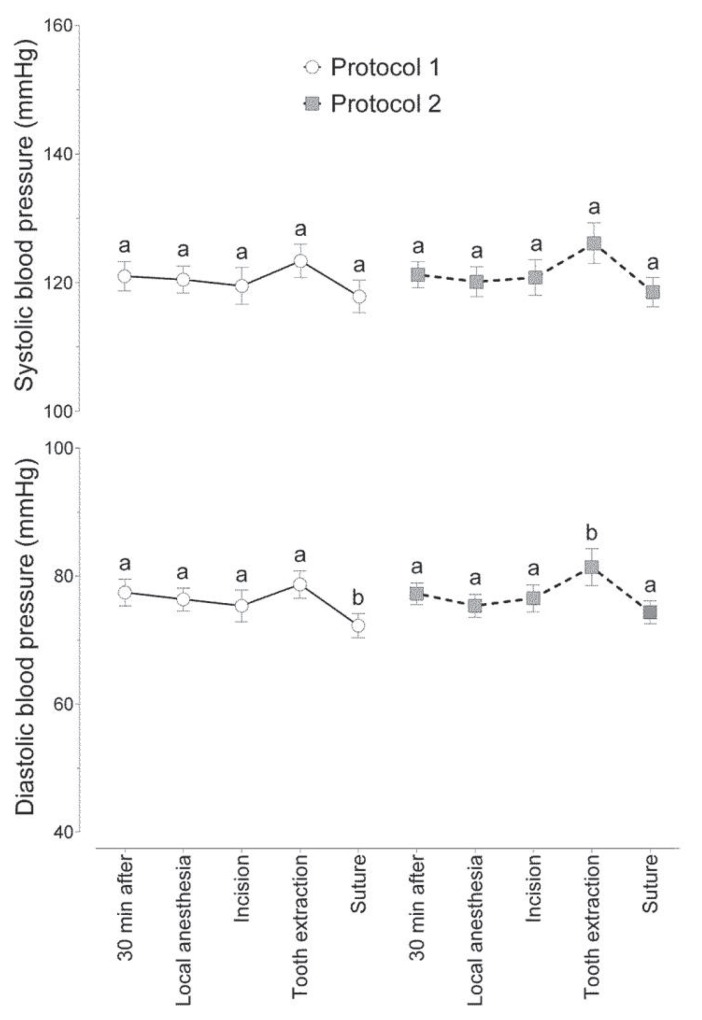
Mean (± standard error of the mean) systolic and diastolic blood pressure at each observation period for 2 protocols. Different letters indicate statistically significant differences between observation periods within the same protocol.

Figure 1 shows the influence of both protocols on maximum and minimum BP. No statistically significant difference (2-way ANOVA with Holm-Sidak correction for multiple comparisons, *p* = 0.0928) was found between the protocols or between the observation periods for systolic BP. However, the diastolic pressure showed a statistically significant difference (2-way ANOVA, *p* = 0.0132), as shown in figure. 1. The minimum values ​​for BP observed during extraction were higher than values observed during local anesthesia, incision, and suturing, but not different from baseline (30 minutes after administration) for Protocol 2 (*Passiflora*). Considering only Protocol 1 (midazolam), suturing had lower minimal BP compared to the initial period and extraction. There were no statistically significant differences between protocols (*p* > 0.05), considering each surgical time point independently.

**Figure 2:**
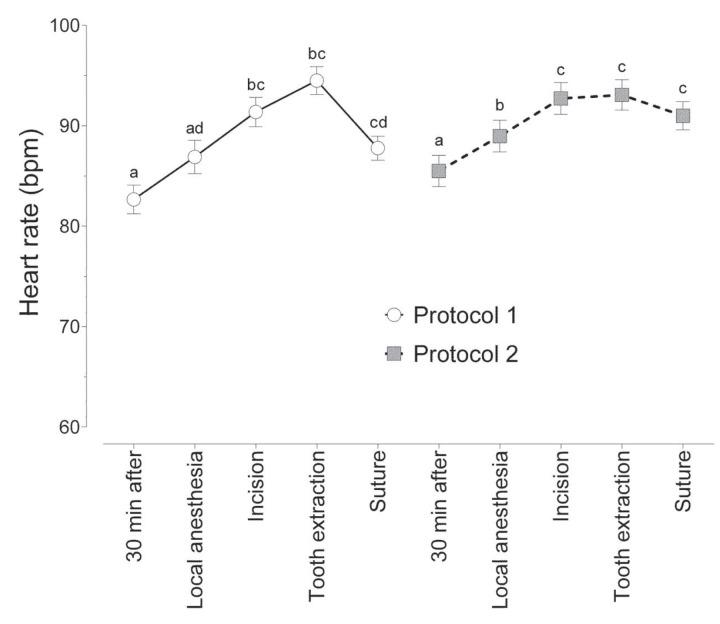
Mean (± standard error of the mean) heart rate at each observation period for 2 protocols. Different letters indicate statistically significant differences between observation periods within the same protocol.

For both protocols, HR increased during extraction and remained higher than the initial rate until suturing (Fig. 2). There were no statistically significant differences between the protocols in any of the operative periods.

**Figure 3:**
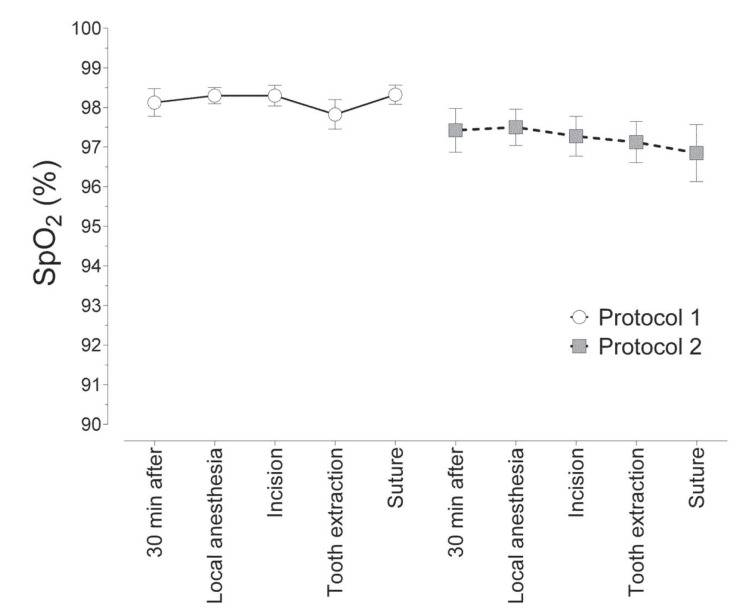
Mean (± standard error of the mean) arterial oxygen saturation (SpO2) at each observation period for 2 protocols.

The SpO2 shown in figure 3 was not significantly different (2-way ANOVA, *p* = 0.0633) between observation periods or between protocols.

Were analyzed the results of evaluation of the degree of anxiety by the researcher and the operator on the day of the procedure. No statistically significant difference (chi-square test, *p* > 0.05) was found between protocols. Moreover, no statistically significant difference (chi-square test, *p* > 0.05) was found between evaluations conducted by the researcher and by the operator.

 Table 2 shows the results of self-assessment of anxiety level and considers the order in which the operations were performed. It was possible to observe an effect of Protocol 1 (midazolam) in the second surgery, because it increased the proportion of participants who reported that they felt quiet. We observed the opposite effect for Protocol 2 (*Passiflora*). In other words, participants who underwent the second surgery with Protocol 2 were more likely to be anxious. This result may be linked to the data in table 3 showing that participants receiving Protocol 2 were more likely to remember the surgery.

A significantly greater proportion (Fisher exact test, *p* < 0.0001) of participants reported some interference with memory for Protocol 1 than for Protocol 2. The results showed that Protocol 2 (*Passiflora*) showed little or no ability to interfere with memory formation ( Table 3).

**Table 2:**
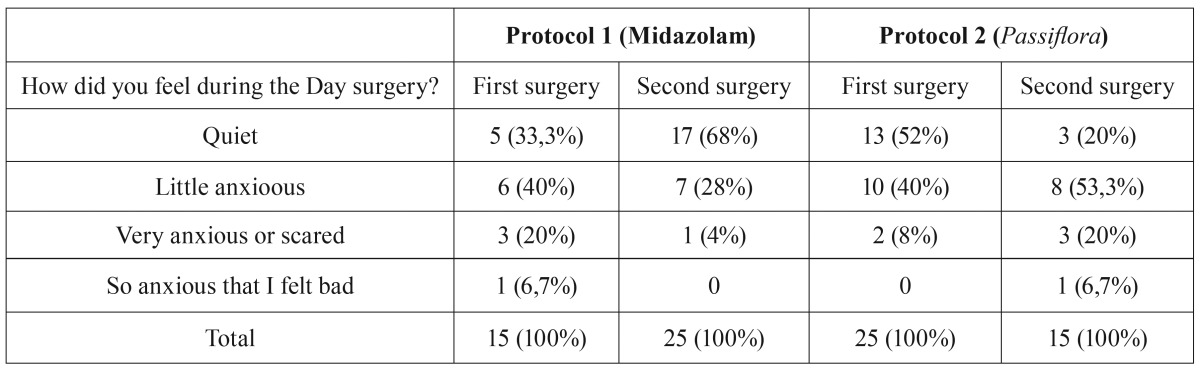
Proportion of responses to the question, “How did you feel during the day of surgery?”

**Table 3:**
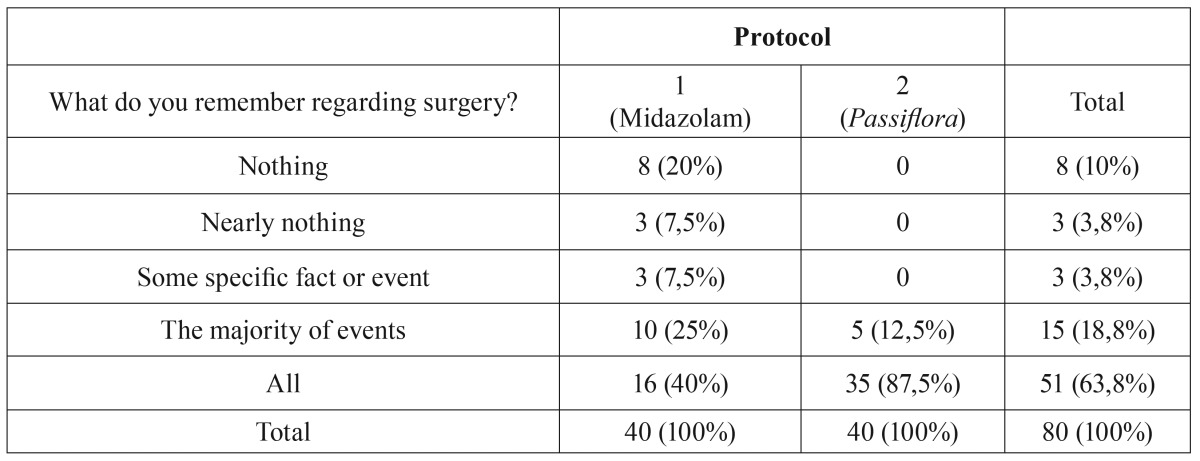
Interference of the drugs with participants’ memories.

 Table 4 shows the adverse effects reported by participants for each protocol. Drowsiness was the most frequently reported effect. There was no statistically significant difference (chi-square test, *p* = 0.0863) between the protocols in relation to adverse effects.

**Table 4:**
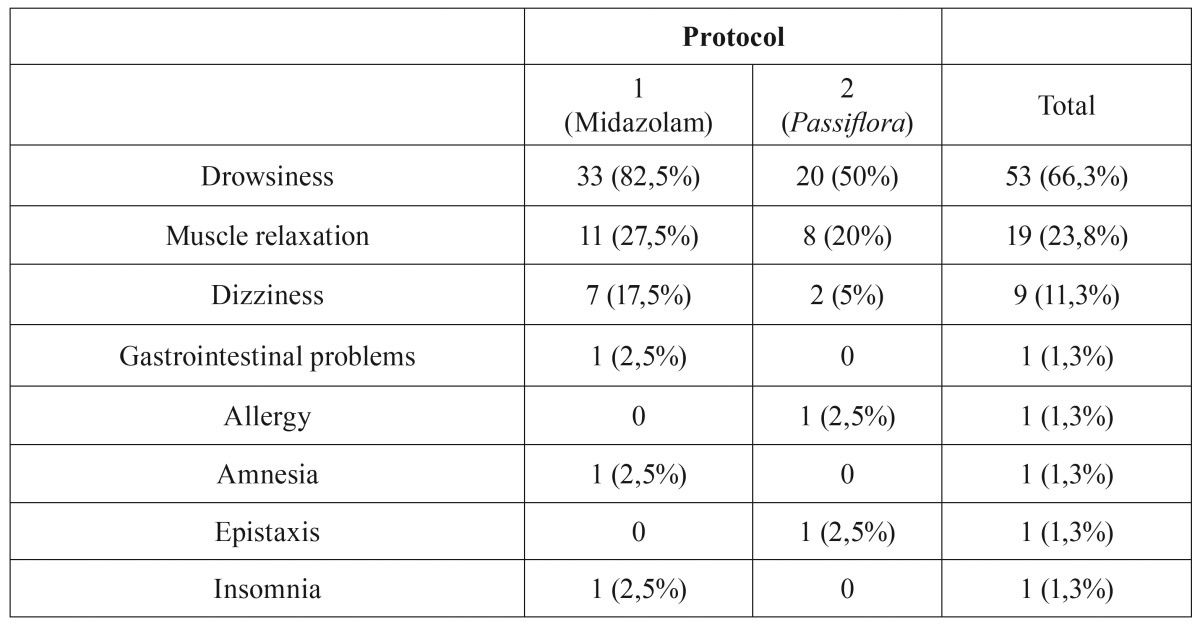
Adverse effects reported by participants for each protocol.

Regarding the relative preference of the participants for each protocol, twenty-one of 40 subjects (52.5%) preferred Protocol 1 (midazolam) at either the first or the second surgery, whereas 11 participants (27.5%) preferred Protocol 2 (*Passiflora*), and 8 participants (20%) did not report a preference for either protocol.

## Discussion

To compare the anxiolytic activity of *Passiflora incarnata* with midazolam, we opted for an experimental model incorporating bilateral extraction of the mandibular third molars. Participants served as their own controls in this double-blind crossover study.

Anxiety is an inevitable component of oral surgical procedures. Sedation is an effective method to reduce anxiety, and midazolam is widely used for this purpose because it provides profound anterograde amnesia and satisfactory sedation with minimal changes in the cardiovascular and respiratory systems. Midazolam has become the standard drug because it is an ideal sedative for this type of surgical procedure. Its short action is consistent with the average duration of a dental procedure (5-7,13-18). However, contrary to most authors, Zanette *et al.* (19) concluded in their work that diazepam would be better than midazolam in conscious sedation dentistry.

In this study, 52.5% of the sample consisted of low-anxiety individuals, a result similar to that of Campos *et al.* (2) who reported a prevalence of 47.6%. Results of this study showed a prevalence of 2.5% for extremely anxious individuals, considerably divergent from the results of 12.3% reported by Campos *et al.* (2).

Our results showed that women had higher levels of anxiety than men. However, Campos *et al.* (2) did not find a significant association between anxiety and gender, but did corroborate that age did not influence anxiety levels.

No statistically significant difference was found between the protocols for systolic BP. However, measurement of diastolic pressure showed a significant difference. Under Protocol 2 (*Passiflora*), the minimum BP values​ during extraction were higher than those observed during other time points (local anesthesia, incision, and suturing), but did not differ significantly from baseline values. This can be explained by the fact that extraction is the most stressful moment during the surgical procedure.

Under Protocol 1 (midazolam), lower diastolic BP values were noted during suturing than during the initial period and extraction. Participants may have felt relieved when the tooth removal was completed and, thus, were able to relax more effectively. However, no statistically significant differences were found between protocols for BP, when each operative period was considered independently. This result shows that *Passiflora* and midazolam have similar anxiolytic activity.

Our results showed that HR increased during extraction and remained higher than initial levels until suturing for both protocols. There were no significant differences between the protocols in any of the operative periods, showing that the drugs had a similar effect on HR during third molar extraction.

Respiratory depression is a common collateral effect of benzodiazepines. Various levels of respiratory depression have been reported, according to the dosage and type of sedative medication. Midazolam is accepted as a safe sedative agent with a minimal incidence of adverse effects. Although this study was not placebo-controlled, considering the favorable SpO2 value for a healthy individual to be between 99% and 95%, our results showed that midazolam and *Passiflora* did not significantly change SpO2 at the doses used in this study. *Passiflora* does not produce respiratory depression, making it safe for clinical use. In accordance with our findings, Ustun *et al.* (5) and Fan *et al.* (17) reported that midazolam preserved SpO2 throughout surgery.

Anterograde amnesia (forgetting information that is acquired after drug administration) has been demonstrated following administration of midazolam in several studies (5,6,14,20-23). Amnesia seems to be an advantage for patients who want to avoid the memory of an unpleasant experience during dental surgery. In our study, 20% of the participants who received midazolam reported not remembering anything. However, amnesia was not reported by any participant who received *Passiflora*. Midazolam was associated with a significantly higher proportion of patients with altered memory than *Passiflora*. Accordingly, *Passiflora* showed little or no ability to interfere with memory.

Several studies have reported somnolence as a common adverse effect of midazolam. In a study by Ritwik *et al.* (18), 66.7% of the subjects showed somnolence after oral administratio n of midazolam. In the present study, somnolence was the most commonly reported adverse effect for both protocols, reported by 82.5% and 50% of participants who received midazolam and *Passiflora*, respectively.

Finally, the participants were asked which surgery felt better. Fifty-two percent of participants preferred the surgery with midazolam, while 27.5% preferred the surgery with *Passiflora*, and 20% felt there was no difference between the protocols. The preference for midazolam can be explained by the fact that this drug has the ability to cause anterograde amnesia, resulting in participants not remembering what happened in surgery and eliminating possible formation of negative memories of the procedure. Memory interference was the only variable resulting in a statistically significant difference between the protocols; therefore, it may be the reason participants preferred midazolam.

Since this is the first study assessing the anxiolytic activity of *Passiflora incarnata* in surgical dental procedures, it is difficult to discuss the present results in a broader context. We believe, however, that *Passiflora* showed anxiolytic activity similar to midazolam, with good tolerability, and may constitute an important pharmacological alternative for the management of anxiety during dental treatment.

We concluded from the results of this research that *Passiflora incarnata* has an anxiolytic effect when administered in the preoperative oral dose of 260 mg, and is safe and effective for conscious sedation in adult patients undergoing extraction of the mandibular third molars. Comparing the anxiolytic effect of *Passiflora incarnata* (260 mg) with midazolam (15 mg) revealed the similarity between the drugs. Participants receiving either drug maintained a relatively stable BP and HR, with slight variations according to the stages of the surgery, but without exceeding the limits of normality.
